# Takotsubo Syndrome and Cancer: Pathophysiological Links and Clinical Perspectives

**DOI:** 10.3390/biomedicines13112718

**Published:** 2025-11-06

**Authors:** Adriana Correra, Alfredo Mauriello, Anna Chiara Maratea, Celeste Fonderico, Matilde Di Peppo, Vincenzo Russo, Antonello D’Andrea, Giovanni Esposito, Natale Daniele Brunetti

**Affiliations:** 1 Cardiology Department, Ospedali Riuniti University Hospital, Viale Pinto 1, 71122 Foggia, Italy; matilde.dipeppo@gmail.com (M.D.P.); natale.brunetti@unifg.it (N.D.B.); 2 Complex Structure Cardiology, Institute National Cancer, IRCCS, Foundation “G. Pascale”, 80131 Naples, Italy; alfredo.mauriello93@libero.it (A.M.); annachiara.maratea@gmail.com (A.C.M.); celeste.fonderico@istitutotumori.na.it (C.F.); 3 Cardiology Unit, Department of Medical and Translational Sciences, “Monaldi Hospital”, 80131 Naples, Italy; vincenzo.russo@unicampania.it; 4 Cardiology and Intensive Care Unit, Department of Cardiology, Umberto I Hospital, 84014 Nocera Inferiore, Italy; antonellodandrea@libero.it; 5 Department of Advanced Biomedical Sciences, University of Naples Federico II, 80131 Naples, Italy; giovanni.esposito2@unina.it

**Keywords:** Takotsubo syndrome, cancer, chemotherapy, immune checkpoints inhibitors, VEGF inhibitors, fluoropyrimidines

## Abstract

Takotsubo syndrome (TTS) is an acute, reversible cardiomyopathy that clinically mimics acute coronary syndrome in the absence of obstructive coronary artery disease. In oncology, TTS may be precipitated by the cancer milieu itself (stress, inflammation, neuroendocrine activation) and by antineoplastic therapies, notably fluoropyrimidines, vascular endothelial growth factor (VEGF) pathway inhibitors, tyrosine kinase inhibitors, and immune checkpoint inhibitors. Cancer currently stands as the second leading cause of morbidity and mortality worldwide. Cancer can directly induce TTS through an increase in catecholamines or indirectly via surgical and chemotherapeutic treatments. Several antineoplastic drugs are associated with an increased risk of TTS. We conducted a narrative, clinically oriented review. This narrative review aims to analyze the pathophysiological link between TTS and cancer and to explore potential preventive and therapeutic strategies in cancer patients.

## 1. Introduction

**Figure 1:**
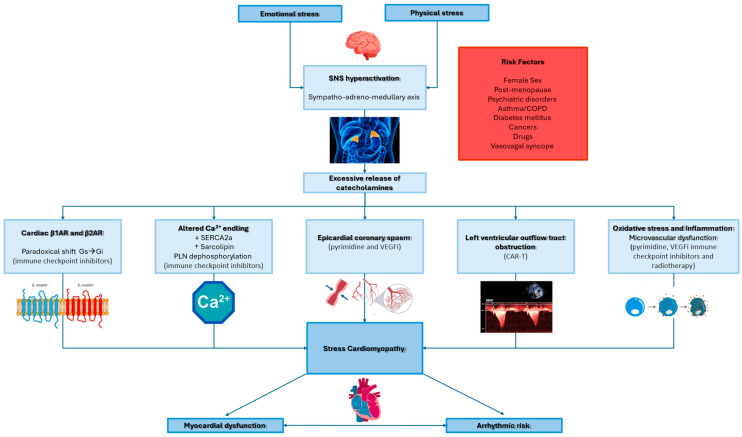
Proposed mechanisms linking cancer and Takotsubo syndrome: catecholamine surge, receptor signaling changes (β1/β2; G-protein switching), microvascular dysfunction, oxidative stress/inflammation, and treatment-related triggers (fluoropyrimidines, VEGF/TKI agents, ICIs). β1AR: beta-1 adrenergic receptor; β2AR: beta-2 adrenergic receptor; COPD: chronic obstructive pulmonary disease; Gi: inhibitory guanine nucleotide-binding proteins; Gs: stimulatory guanine nucleotide-binding proteins; LVOTO: left ventricle outflow tract obstruction; PNL: phospholamin; SERCA2a: sarco-endoplasmic reticulum calcium ATPase 2a; SNS: sympathetic nervous system. VEGF: vascular endothelial growth factor. ↑: increasing; ↓: decreasing.

Takotsubo syndrome (TTS) is an acute and reversible cardiomyopathy that may present with a clinical presentation like acute coronary syndrome (ACS) [1] in the absence of non-obstructive coronary atherosclerotic disease [2]. TTS prevalence accounts for 2–3% of patients presenting with ACS [3] and increases to 5–6% in female patients [4]. A multimodality approach is often needed for the differential diagnosis with other acute cardiac presentations [5]. There are several pathophysiological hypotheses on the underlying mechanisms of TTS [6]; however, the activation of the sympathetic nervous system, triggered by emotional or physical stress, with massive release of catecholamines, seems to be the most plausible [7,8]. Cancer is a disease of uncontrolled proliferation by transformed cells subject to evolution by natural selection [9]. Incidence rates in the 50 selected registries range from over 400 per 100,000 males and 300 per 100,000 females to less than 100 per 100,000 in both males and females. Mortality rates in the 50 selected countries range from over 200 deaths per 100,000 males and over 100 deaths per 100,000 females to less than 50 deaths per 100,000 in both males and females. For both sexes, the highest rates are generally in North America, Oceania, and Europe [10]. Cancer is associated with several complications, including cardiac manifestations [11]. The term “cardiotoxicity” refers to the consequences of cancer treatment [11]. Among the cardiological manifestations, there is TTS. The prevalence of cancer in TTS is 10%, with a range of 4% to 29% [12]. TTS can be triggered both by cancer itself, through physical and psychological stress, and through surgical and pharmacological treatments. Our comprehensive review aims to define the pathophysiological relationships between TTS and cancer, which chemotherapy drugs can increase the risk of TTS, and what preventive strategies can be adopted in patients with cancer to prevent TTS. Figure 1 summarizes proposed mechanisms linking cancer and TTS [13].

## 2. Materials and Methods

This article is conceived as a narrative, clinically oriented review regarding the state of the art about the relationship between TTS and cancer. Its primary aim is to establish the pathophysiological link between TTS and cancer and to explore potential preventive and therapeutic strategies in cancer patients with a pragmatic synthesis of evidence and expert guidance, rather than to produce a formal systematic review or meta-analysis.

To ensure transparency in the selection of sources, we utilized a structured search strategy that guided, but did not solely determine, the narrative synthesis. A comprehensive search was conducted in PubMed/MEDLINE and EMBASE for articles published from January 2010 to September 2025, employing combinations of the following keywords and Boolean operators: “Takotsubo” OR “Takotsubo Syndrome” AND (“cancer” OR “cardiotoxicity” OR “biomarkers” OR “imaging” OR “multidisciplinary care” OR “inflammation”). The search was limited to English-language publications with available abstracts.

Notably, we included clinical studies, prospective and retrospective cohorts, randomized controlled trials, meta-analyses, and relevant registry reports addressing the risk of TTS in cancer patients.

We excluded narrative reviews, case reports, editorials, commentaries, experimental pre-clinical studies without direct clinical implications, and conference abstracts without full data.

Although the review is not systematic and no pooled quantitative synthesis was performed, we assessed the quality of included evidence by considering key domains relevant to clinical studies: patient selection (representativeness of study populations, inclusion/exclusion criteria); outcome ascertainment and follow-up (definition and measurement of cardiotoxicity or clinical endpoints); and control for confounding (adjustment for baseline cardiovascular risk factors, treatment exposure, and competing risks). This qualitative appraisal was informed by the Joanna Briggs Institute (JBI) Critical Appraisal Checklists for observational studies [14] and, when applicable, by the Cochrane Risk-of-Bias framework for randomized trials. These tools were used to guide judgment on the relative weight assigned to different types of evidence in the discussion.

Although we employed a reproducible search strategy for transparency, we did not adopt a full PRISMA methodology, including duplicate screening, formal scoring of risk of bias, or meta-analytic pooling, because of the heterogeneity of study designs (clinical trials, registries, organizational models, and policy analyses) and the practical focus on management and health-system implementation. A formal PRISMA-based systematic review was therefore considered inappropriate for the present purpose. To make this distinction explicit, we state that the search results were used to inform a structured narrative synthesis, not to support a systematic review. Wherever feasible, we specify in the text whether the conclusions derive from meta-analyses, multicenter studies, or smaller observational series, thereby enabling the reader to gauge the robustness of the evidence base.

## 3. Physiopathological Mechanisms of Takotsubo Syndrome

TTS is a condition whose pathophysiology is not yet fully understood, but current research suggests several key mechanisms. The most widely accepted hypothesis suggests that TTS is triggered by a surge of catecholamines, often following intense emotional or physical stress [15]. When a person experiences a significant stressor, their sympathetic nervous system is activated, releasing a large amount of catecholamines. This excess of hormones is thought to be a primary driver of TTS [16]. These high levels of catecholamines can damage the heart muscle in several ways [17]. Catecholamines can be directly toxic to heart cells, especially at the apex of the left ventricle (LV), which has a higher density of certain receptors that respond to these hormones [16,18,19]. Extremely high levels of epinephrine can paradoxically weaken heart contractions by altering the signaling pathways of beta-adrenergic receptors [20,21]. The activation of beta-adrenergic receptors can also lead to the production of harmful free radicals, causing oxidative stress and damage to the heart muscle [22,23]. While catecholamines are central to TTS, other factors may also contribute to the condition. The sudden release of catecholamines can lead to coronary artery spasms [24] and microvascular dysfunction [25], causing myocardial ischemia. However, this is likely a secondary effect rather than the primary cause, as perfusion defects in the heart often appear after cardiac dysfunction has already begun. In some cases, TTS is associated with an obstruction in the LV’s main outflow tract (LVOTO), which can cause apical subendocardial ischemia [26,27]. However, this is not seen in all TTS patients and is likely a complication rather than a central cause. Oxidative stress may impact TTS pathophysiology by endothelial dysfunction, which is caused by the disruption of endothelial nitric oxide synthase (eNOS), an enzyme essential for maintaining vascular function. Inflammation is another key factor in the pathophysiology of TTS [28]. Elevated levels of C-reactive protein (CRP) and increased leukocyte counts, markers of systemic inflammation, are commonly observed in TTS patients [29].

The TTS pathophysiology is complex, and while a catecholamine surge appears to be the main trigger, other factors contribute to the full clinical picture. The transient nature of the condition and its rapid recovery with supportive care further highlight the unique mechanisms at play.

## 4. Specific Triggers in Cancer Patients

The link between cancer and TTS is complex and often explained by a “multi-hit hypothesis”. Emotional and psychosocial stress: emotional trauma related to a cancer diagnosis, a poor prognosis, or generalized emotional disorders affects nearly half of cancer patients and acts as a significant trigger [30]. Chronic inflammatory state: the inflammatory state induced by malignancy itself and circulating paraneoplastic mediators are considered risk factors. In REgistry on TAKOtsubo Syndrome (RETAKO), 463 patients were included (median age 73 years, 13% men), and higher levels of heart failure (HF) and inflammatory markers such as N-terminal pro-B-type natriuretic peptide (NT-proBNP) and high-sensitivity CRP (hs-CRP) were identified as predictors of late recovery [16.0% vs. 8.6%, adjusted hazard ratio (HR): 1.31; 95% confidence interval (CI): 1.12–1.60] and increased severity in TTS patients with cancer (adjusted HR: 1.08; 95% CI: 1.04–1.13) [31]. Physical stress resulting from cancer surgery, radiation treatment, acute illness, pain crises, or systemic antineoplastic therapy can trigger TTS. In a meta-analysis of Brunetti et al., including 123,563 patients, physical stressors were reported to be more prevalent in TTS patients with cancer (58%) compared to controls (44%) (*p* = 0.0058) [32]. Certain tumors, such as those of neuroendocrine origin, can secrete large quantities of catecholamines, which may be responsible for the onset of TTS [33].

## 5. Chemotherapy and Takotsubo Syndrome

Numerous antineoplastic agents, including both traditional chemotherapy and targeted drugs, have been associated with TTS [34]. The proposed mechanism between antineoplastic drugs and TTS is described in Table 1.

### 5.1. Fluoropyrimidines

5-Fluorouracil (5-FU) is the drug most frequently implicated in triggering TTS. Cardiotoxicity related to 5-FU may manifest as TTS, often occurring during or immediately after continuous infusion, commonly in the first cycle (70% of cases) [35]. Unlike TTS in the general population, 5-FU-related TTS shows a similar incidence in both sexes. Capecitabine, an oral prodrug of 5-FU, has also been linked to TTS, usually occurring during the first cycle of treatment [36].

### 5.2. Targeted Agents

These include vascular endothelial growth factor (VEGF) inhibitors (e.g., bevacizumab) [37] and tyrosine kinase inhibitors (TKIs), such as sunitinib and axitinib [38], and CAR-T cell therapy [39].

### 5.3. Immune Checkpoint Inhibitors

TTS is a recognized, although relatively rare, immune-related cardiovascular adverse event (irCVE) in patients receiving ICIs (ipilimumab, pembrolizumab, nivolumab, etc.). An analysis of the WHO global safety database (VigiBase^®^) [38] suggested that ICIs could potentially provoke TTS, with an overall reporting odds ratio (ROR) of 3.39 (95% CI 1.96–5.86, *p* < 0.0001) for all three major ICIs. The most common cancer types in reported ICI-related TTS cases are lung cancer (35%) and melanoma (29%). The median time to TTS onset after ICI initiation was reported as 77 days. Three cases (60%) occurred during the first 6 weeks of treatment.

### 5.4. Other Agents

Some case reports describe the use of rituximab [40] and anthracyclines [41,42] and TTS.

### 5.5. Radiotherapy

Radiation therapy (RT) for cancers in the thoracic/mediastinal region has been linked with heart damage following years of radiation exposure. Desai et al. [43], in a national registry including 5,991,314 hospitalizations with prior intrathoracic/mediastinal malignancies and RT, showed higher odds and increasing trends in TTS-related admissions with worse in-hospital outcomes among patients with prior intrathoracic/mediastinal cancer and RT (*p*_trend_ < 0.001), irrespective of the time interval from cancer diagnosis or RT to TTS occurrence.

The diagnosis of TTS in these patients is challenging due to the potential overlap with other severe cardiotoxicities, particularly myocarditis. Myocarditis requires urgent ICI discontinuation and high-dose corticosteroid administration. Five reported cases had concurrent myocarditis (possible or diagnosed). The diagnostic workup, based on cardiac magnetic resonance (CMR) or biopsy, is vital to exclude myocarditis [44,45]. Therefore, some cancer therapies, such as anthracyclines, can induce a secondary cardiomyopathy, called inflammatory cardiomyopathy [45]. Also in this context, CMR, or, rarely, biopsy, can help clinicians with diagnosis. Figure 1 represents the hypothetical mechanism underlying the relationship between TTS and chemotherapy agents.

Table 1 summarizes physiopathological and pharmacological links between cancer and TTS.

## 6. Clinical Presentation and Diagnosis

### 6.1. Clinical Presentation

The clinical presentation of TTS in cancer patients often mimics acute coronary syndrome (ACS), typically manifesting as chest pain, dyspnea or syncope/pre-syncope [30]. In extreme circumstances, patients can present severe HF, cardiogenic shock, or arrhythmias requiring hemodynamic and sometimes ventilatory support. Patients with cancer can have subtle symptoms [46].

The diagnostic workup involves evaluating laboratory markers and echocardiographic data, as well as ruling out atherosclerotic ACS through prior coronary angiography. Finally, CMR is decisive for a definitive diagnosis. Cancer patients may have alterations dependent on previous cancer therapies.

### 6.2. Echocardiogram

Echocardiography is a cornerstone for the diagnosis of TTS, allowing for the identification of regional and global ventricular wall motion abnormalities in the absence of obstructive coronary artery disease [47]. The key echocardiographic features include specific patterns of dysfunction, reversibility, and the lack of a single vascular territory involvement. The most unique and common finding in TTS is ventricular “ballooning.” This phenomenon, which gives the syndrome its name, manifests as an alteration in the kinetics of the LV. Four main patterns have been described:

Apical ballooning (classic pattern): This is the most frequent form, accounting for over 80% of cases. It is characterized by hypo-akinesis or dyskinesis of the apical and mid-ventricular segments. This is accompanied by a compensatory hyperkinesis of the basal segments, which keeps the left ventricular ejection fraction (LVEF) in a variable range, although it is often markedly reduced in the acute phase [48].

Mid-ventricular ballooning: A less common pattern, where akinesis or hypokinesis is localized to the mid-ventricular segments of the LV, with normal or hyperkinesis of both the apical and basal segments [48,49].

Reverse TTS (or basal ballooning): In this pattern, hypokinesis or akinesis affects the basal segments of the LV, while the apex and mid-segments show normal or increased contractility. This is a rarer finding and is more often seen in younger patients and those with physical triggers [50].

Focal TTS: A very rare pattern, where dysfunction is localized to a single area of the myocardium, without the widespread involvement typical of the other patterns [51].

In about a quarter of cases, echocardiography may also reveal an extension of the ballooning to the right ventricle (RV), with hypo-akinesis of the apical and mid-segments. This combined (biventricular) involvement further supports the diagnosis of TTS [52].

Furthermore, the peculiar hyperkinetic motion of the basal segments can lead to dynamic LVOTO. The exaggerated systole of the base can cause an acceleration of blood flow that “pulls” the anterior leaflet of the mitral valve toward the interventricular septum. This phenomenon, known as systolic anterior motion (SAM), is associated with an intraventricular pressure gradient and can lead to mitral regurgitation, as well as, in severe cases, cardiogenic shock [39].

Another fundamental echocardiographic feature of TTS is the complete reversibility of ventricular dysfunction. Follow-up echocardiography, typically within a few days or weeks, shows a gradual and complete recovery of LVEF and segmental motion [53]. This is an important finding for differentiating TTS from a myocardial infarction.

Finally, the segmental wall motion abnormalities in TTS do not correspond to the distribution territory of a single coronary artery. This finding, combined with coronary angiography, is pathognomonic for TTS [54].

### 6.3. Electrocardiogram

Patients usually exhibit new electrocardiogram (ECG) abnormalities, such as ST-segment deviation, T-wave inversion and QTc interval prolongation [55]. ST-segment elevation is the most frequent finding in the acute phase, occurring in about 40% of cases. Unlike ST elevation myocardial infarction (STEMI), where ST elevation is typically confined to a specific vascular territory, in TTS, the elevation tends to be more diffuse, typically involving precordial and inferior leads [56]. Corrected QT-interval (QTc) prolongation is a frequent finding and can persist for weeks or months. This finding is of clinical relevance as it increases the risk of malignant ventricular arrhythmias, such as torsades de pointes [57]. Unlike in STEMI, the development of pathological Q-waves is rare in TTS, suggesting the absence of transmural necrosis [58]. In some cases, a reduction in the amplitude of the R-wave in the precordial leads may be observed, but this is not a specific diagnostic criterion [58]. The absence of pathological Q-waves helps to distinguish TTS from a myocardial infarction [58]. Table 2 summarizes different ECG features between STEMI and TTS.

### 6.4. Biomarkers

Cardiac enzymes (troponin) show a mild elevation, disproportionately low compared to the extent of myocardium affected [59]. NT-proBNP levels are typically elevated, correlating with the degree of ventricular dysfunction [60]. In cancer patients, these markers may be increased due to other causes [60].

### 6.5. Coronary Angiography and Ventriculography

Coronary angiography (CA) must show the absence of obstructive coronary disease or acute plaque rupture [61]. The most distinctive and pathognomonic angiographic feature of TTS is the normality or absence of significant coronary lesions (>50% stenosis) that could justify clinical presentation. Although patients with TTS may occasionally have insignificant atherosclerotic plaques, CA does not reveal an acute obstruction caused by a thrombus or a critical stenosis in one of the epicardial arteries that could explain the ventricular wall motion abnormalities observed on the echocardiogram. This suggests a possible microvascular dysfunction that contributes to the transient myocardial dysfunction [62].

**Figure 2:**
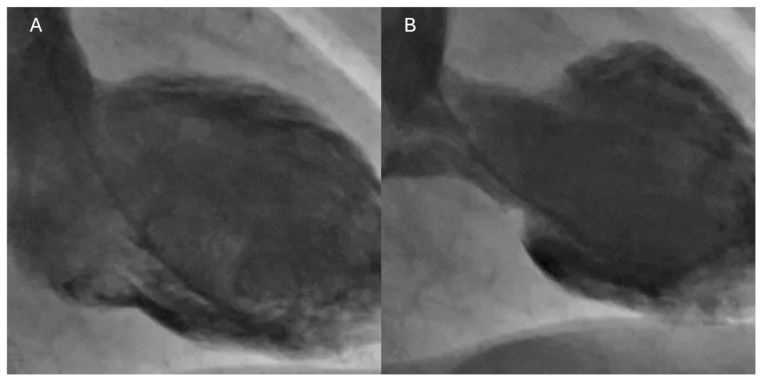
Left ventriculography of apical ballooning TTS. (**A**) End-diastolic phase; (**B**) End-systolic phase.

During CAG performed during the acute phase, left ventriculography is an invasive diagnostic test used to determine ventricular function. It is useful for excluding or confirming TTS [63]. Left ventriculography allows the evaluation of LV function and morphology, identifying the typical TTS patterns when echocardiography is not available or when wall motion abnormalities cannot be properly assessed with ultrasound [47]. Figure 2 represents typical apical ballooning TTS during left ventriculography.

### 6.6. Cardiac Magnetic Resonance

In oncology patients, the differential diagnosis is critical. It must exclude ACS, via CAG, and myocarditis, especially relevant for ICI-treated patients. The differentiation between TTS and other chemotherapy-related cardiac dysfunction (CTRCD) or immune-related myocarditis is often difficult, particularly if LV dysfunction is diffuse. For hemodynamically stable patients, CMR is the preferred non-invasive modality to exclude myocarditis, though endomyocardial biopsy remains the definitive test for unstable patients [45].

Similar to echocardiography, CMR allows for an accurate and reproducible evaluation of left and right ventricular function and wall motion. Cine-CMR images confirm the characteristic patterns of regional dysfunction already described. Therefore, tissue characterization in TTS is superior to echocardiography for differential diagnosis. Tissue characterization techniques include:

- T2-weighted imaging: In the acute phase of TTS, myocardial hyperemia and inflammation caused by excess catecholamines lead to an increase in tissue water content. T2-weighted images show myocardial edema that typically extends along the dyskinetic segments. This edema is a sign of acute and transient injury [64].
- Late gadolinium enhancement (LGE): LGE is the standard technique for identifying myocardial fibrosis or irreversible necrosis. In TTS, the characteristic finding is the absence of LGE in the segments with ventricular dysfunction. This absence is an essential differentiator from myocardial infarction, where LGE is present in a subendocardial or transmural pattern corresponding to the territory of an occluded coronary artery. The absence of LGE in TTS reflects the lack of irreversible necrosis, supporting the benign and transient nature of the condition [65].
- T1 mapping and T2 mapping: These quantitative techniques offer a more sensitive and specific assessment of diffuse edema and fibrosis. In acute TTS, T2 mapping values are elevated, reflecting the edema, while T1 mapping values are typically normal or only slightly elevated [66]. Table 3 summarizes the different features between TTS and ICI myocarditis.

### 6.7. Prognosis

Cancer-associated TTS is associated with higher in-hospital adverse events than TTS without cancer. Delayed recovery of LV function (≥10 days) portends worse short- and long-term outcomes. TTS patients with coexisting malignancy face significantly worse outcomes. In a retrospective study that aimed to evaluate the association between the use of TTS and cancer survival rate, in-hospital mortality is substantially higher in TTS patients with cancer (12.8% to 13.8%, *p* < 0.05) compared to those without malignancy (2.9% to 3.8%, *p* < 0.05). The overall risk of clinical events at follow-up is also higher in the cancer group (RR 3.24). Factors associated with higher mortality include solid cancer (OR 3.43) and hematological cancer (OR 3.21) [67]. LV recovery is defined as normalization of left ventricular ejection fraction (LVEF > 50%, or return to a previously reported value) together with complete resolution of regional WMA present at admission. LV recovery is usually achieved within days to weeks. However, recovery is defined as late if it occurs 10 days or more after the index event. Late LV recovery is associated with worse short- and long-term survival [68].

Predictors of late recovery, in a multivariate analysis, are independent predictors including older age, history of neurological disorders, active cancer, physical triggers, bystander coronary artery disease (CAD), cardiogenic shock, lower LVEF at admission, and elevated inflammatory markers (NT-proBNP and hs-CRP) [69]. Therefore, the risk of death associated with late LV recovery increases exponentially, rising by 8% for every additional 10-day delay in time-to-LV recovery [70]. Non-cardiovascular and cardiovascular comorbidities are the main determinants of outcome in TTS, especially in the long term [71].

### 6.8. Therapeutic Management

Initial management involves supportive care, focusing on managing complications such as HF and cardiogenic shock [72].

For hemodynamically stable patients, the initiation of guideline-directed medical therapy (GDMT) for HF with reduced ejection fraction (HFrEF) is the mainstay, including beta-blockers and ACE inhibitors (or ARBs) [73,74]. If LVOTO obstruction is present, positive inotropic agents, such as dopamine and dobutamine, and vasodilators should be avoided, as they can worsen the obstruction. Fluids, β-blockers, and vasoconstrictors should be considered as appropriate [75,76,77].

Due to apical akinesia, LV apical thrombosis is a possible complication in up to 4% of cases; in such cases, anticoagulation should be considered [78]. In a multicenter study [79] including 541 TTS patients, Santoro et al. found that 12 patients (2.2%) developed LV thrombi (all female presenting with apical ballooning pattern). Among these patients, 2 out of 12 (17%) had a stroke before anticoagulation initiation. These patients were characterized by a high prevalence of ST elevation and higher troponin I levels. Troponin I levels > 10 ng/mL were the only predictor of LV thrombosis (normal values = 0.5 ng/mL) [80]. However, the risk/benefit ratio must be considered in cancer patients, as they may be at both high thromboembolic and high bleeding risk. Therefore, the decision to initiate anticoagulant therapy in these patients should be individualized [80].

Regarding the reintroduction of oncologic therapy, it is a clinical challenge. If TTS is induced by ICIs, ICI treatment is generally stopped. For other antineoplastic drugs, some patients have safely resumed treatment after LVEF normalization, which typically takes about 20 days [81].

## 7. Future Perspective

The co-occurrence of TTS and malignancy, particularly in the context of advanced antineoplastic treatments like ICIs, presents significant diagnostic and therapeutic challenges. Future research and clinical efforts should focus on the following priorities:

1. Pathophysiological distinction and diagnostic refinement: Further studies are required to definitively determine if cardiotoxicity induced by oncology drugs, especially ICIs, represents a true TTS, ICI-related myocarditis, or a distinct type of chemotherapy-related reversible non-ischemic cardiomyopathy. It is important to implement early diagnostic algorithms, including CMR and endomyocardial biopsy, to distinguish TTS from myocarditis in both clinically stable and unstable patients.
2. Risk stratification and surveillance: Identifying cancer patient cohorts at the highest risk of TTS and late LV recovery (e.g., those with active malignancy, physical triggers, and high inflammatory markers) is paramount. Prospective baseline screening using biomarkers, such as troponin and neuropeptides, and cardiac imaging in high-risk patients may enable targeted cardioprotection strategies.
3. Risk of recurrence: Most studies and registries report a recurrence rate that varies approximately between 1% and 6% of patients who have had a first episode. Recurrence can occur at variable time intervals, even several years after the initial event. Some studies suggest that the presence of neurological disorders and psychiatric disorders may be associated with a higher risk of recurrence, but there is no evidence regarding the role of cancer [82].
4. Optimal management and re-challenge safety: There is an unmet need to establish the optimal management of ICI-related TTS, including the definitive role of immunosuppressive therapy, such as corticosteroids, when myocarditis is excluded. Furthermore, definitive data regarding the safety and timing of reintroducing antineoplastic therapy, especially ICIs, after an episode of TTS are urgently needed. Decisions must currently be individualized by a multidisciplinary cardio-oncology team.
5. Prospective studies: Large, prospective multicenter registries are essential to gather high-granularity data on the long-term prognosis and true incidence of TTS in cancer survivors, particularly focusing on the adverse impact of delayed LV functional recovery.

## 8. Conclusions

TTS is a form of ACS generally triggered by either a physical or psychological stressor. The co-occurrence of TTS and cancer presents significant diagnostic and therapeutic challenges, particularly with the rise in advanced antineoplastic treatments like ICIs.

CMR and endomyocardial biopsy to accurately differentiate between TTS, ICI-related myocarditis, and other forms of chemotherapy-related cardiomyopathy. Better risk stratification is essential. Identifying cancer patients at the highest risk for developing TTS, such as those with active malignancy, physical triggers, and high inflammatory markers, is paramount. Prospective screenings using biomarkers such as troponin and natriuretic peptides could aid in implementing targeted cardioprotection strategies for these high-risk individuals.

Additionally, establishing optimal management practices for ICI-related TTS is an urgent priority. This includes determining the definitive role of immunosuppressive therapies, such as corticosteroids, in cases where myocarditis has been ruled out. Furthermore, we need clear, definitive data on the safety and ideal timing for reintroducing antineoplastic therapy after a TTS episode. Currently, these decisions must be made on an individualized basis by a multidisciplinary cardio-oncology team. Finally, large, prospective, multicenter registries are vital to gather high-quality data on the long-term prognosis and true incidence of TTS in cancer survivors, with a particular focus on the negative impact of delayed left ventricular functional recovery.

## Figures and Tables

**Table 1:** Physiopathological and Pharmacological Links Between Cancer and TTS.

Risk Factor/Trigger	Antineoplastic Agents Implicated	Putative Mechanism	Clinical Notes
Emotional/Psychosocial Stress	Immune checkpoint inhibitors	Catecholamine excess, activation of the sympathetic nervous system.	TTS is associated with a higher incidence in females with cancer compared to the general population.
Chronic Inflammation	Rituximab, fluoropyrimidines, VEGF inhibitors and immune checkpoint inhibitors	Release of cytokines/paraneoplastic mediators modifying cardiac adrenoreceptors.	Elevated NT-proBNP and hs-CRP predict late recovery and worse outcomes.
Traditional Chemotherapy Targeted Therapies	5-FU, capecitabine, cisplatin, daunorubicin.	Coronary vasospasm (most common theory for 5-FU), direct myocardial injury.	5-FU-related TTS has a similar incidence across both sexes. Cardiotoxicity often occurs in the first cycle.
	VEGF inhibitors, TKIs, trastuzumab.	Endothelial dysfunction, TKI-related cardiotoxicity, indirect toxicity.	Trastuzumab may induce the “reverse TTS” variant.
	Ipilimumab, pembrolizumab, nivolumab (monotherapy or combination).	Immune-mediated toxicity, overlap with myocarditis, and potentially T-cell cross-reactivity.	ICI exposure suggests a higher risk of TTS. Myocarditis exclusion (via CMR/biopsy) is essential.

5-FU: 5-fluorouracil; hs-CRP: high-sensitivity C-reactive protein; ICIs: immune checkpoint inhibitors; NT-proBNP: N-terminal pro-B-type natriuretic peptide; TKIs: tyrosine kinase inhibitors; TTS: Takotsubo Syndrome; VEGF: vascular endothelial factors.

**Table 2:** Different ECG features between STEMI and TTS.

ECG Features	STEMI	TTS
ST elevation	Regional ST elevation	Diffuse ST elevation
T-wave inversion	Regional T-wave inversion	Diffuse T-wave inversion
QTc evolution	Non-typical	Typical

STEMI: ST elevation myocardial infarction; TTS: Takotsubo syndrome.

**Table 3:** Onco-TTS vs. ICI myocarditis—practical triage.

Features	TTS	ICI Myocarditis
Symptoms/ECG	Diffuse T-wave inversion and QTc prolongation	High-grade AV block
Biomarkers	Troponin pattern	Troponin pattern and inflammatory markers
CMR	Edema in dysfunctional segments with absent or minimal LGE	Typical LGE patterns
Endomyocardial biopsy	If unstable or inconclusive	If unstable or inconclusive
Management	Avoid agents that worsen LVOTO in TTS	Use immunosuppression for suspected ICI myocarditis when appropriate

CMR: cardiac magnetic resonance; ECG: electrocardiogram; LGE: late gadolinium enhancement; LVOTO: left ventricular outflow tract obstruction; TTS: Takotsubo syndrome.

## Data Availability

Not applicable.
